# Typhoid Fever

**Published:** 1837-10-01

**Authors:** 


### MM. BOUILLAUD AND LOUIS ON TYPHOID FEVER.

CLINIQUE MEDICALE DE L'HÔPITAL DE LA CHARITÉ. Par *M. Bouillaud.* Tome 1. Paris, 1837. Bailliere.

ANATOMICAL, PATHOLOGICAL, AND THERAPEUTIC RESEARCHES ON GASTRO-ENTERITE. By *P. C. Louis.* Translated by *H. Bowditch,* M.D. Two Vols. 8vo. Boston, 1836.

THE work, first on our list, is from the pen of that most indefatigable of modern medical authors, Professor Bouillaud, of La Charité Hospital at Paris. It is little more than six months ago since he published an Essay, of some five or six hundred pages, on what he called Medical Philosophy, which our readers will find very copiously reviewed in the number of this Journal for last March. We at that time entered so fully into the merits and demerits of the philosophy of that school, to which M. Bouillaud has most devotedly attached himself, and of which he is by far the most ardent apostle in the present time—so ardent indeed that he may be fairly said to out-Broussais the great reformer himself; and we gave such an ample and withal so candid, we trust, an exposition of the theoretical and practical doctrines of that school, that it would be an utter waste of time to go over the ground again. Yet nothing short of this would be necessary, if we were to attempt to give an analytic review of this Clinique Medicale de l'hôpital de la Charité; for in truth it is only a *riffaciamento* of the contents of the Essay, alluded to above. The ever-recurring themes of the gastro-enteritic origin of continued fevers, of the necessity of repeated venæsections in these and other inflammatory complaints, of the almost constant connexion of pericarditis and endocarditis with rheumatism—these are the subjects on which our author never wearies of ringing eternal changes. A mere glance at the table of contents in the present volume will suffice to convince the reader of the truth of these statements. It consists of 508 pages, of which the first 413 are devoted to the consideration of the various shades and degrees of gastro-enterite; under which term M. Bouillaud treats not only of the different forms of typhus fever, but also of every variety of idiopathic inflammation of the alimentary canal, as well as of most cases of what he calls "embarras gastrique"—in other words, of dyspepsia.

Such is one of the favorite doctrines of the so-called Philosophical School of Medicine in France; that school which, with the most pertinacious dogmatism, would strive to make us believe that almost all diseases are of the same essential nature and origin—viz. of a phlogistic state of the vascular system—and therefore that they all should be treated on one and the same principle. We are not afraid of this very absurd doctrine gaining much ground among the sober doctors of this island; but it is useful nevertheless to make ourselves acquainted with the errors and extravagancies of our flighty neighbours. To justify such a very sweeping censure, our readers may reasonably expect to be furnished with a few proofs; and we shall therefore gratify them with the report of one (we could have selected many others equally pertinent) case of "phlegmasie aigue de l'estomac et des intestines," as a sample of some of the contents of M. Bouillaud's Clinique.

"Observation LXV. A man, 35 years of age, a workman, born at Vannes, indisposed for four days, admitted into the hospital on the 26th August, 1836, discharged on the 31st of August, 1836.

This patient is of a tolerably strong constitution. He came to Paris four days ago (from Bourbon-Vendée); he walked 120 leagues in the space of six or seven days. He has been in the army for ten years; in 1828, he had, according to his own report, a catarrh from the left side of the chest. For four days after his fatiguing journey, he kept quiet in the house. A painful weariness of the body, a swelling of the left foot, and headache then supervening, induced him to apply for admission into the hospital. He complained of pains and a sense of lassitude in the legs, with redness and swelling of the left great toe; he had some headache; the appetite was tolerably good, and the digestive functions were in good condition; there was slight cough; no fever, and the heat of the body was normal. On the 27th, the following day, the report mentions that the patient was "dans le meme etat." He was ordered some vegetable tea, a simple bath, and half the usual allowance of food. On the 31st, he was discharged cured."

Such is the printed description of a case alleged to be illustrative of one of the forms or degrees of acute inflammation of the stomach and intestines!! Is it at all wonderful that a man after a long journey on foot should feel wearied and oppressed? that his legs and feet should be painful and swollen? or that rest in bed for a few days, with the assistance of a tepid bath and some nourishing food, should restore him in four days? Yet this is the exact history of an example of acute inflammation of the alimentary canal, reported in a scientific treatise on clinical medicine by an eminent professor of one of the largest hospitals in Paris in the year 1837!!

We have long suspected M. Bouillaud of exaggeration in all his opinions and statements on medical subjects, and have over and over again expressed our sentiments freely to this effect, more particularly in our late review of his Essay on Medical Philosophy. He is one of those vehement enthusiasts, who mistake the suggestions of their own fancies, or the mere desires of their own wills, for the actual perceptions of their senses, and the deductions of their calm judgment. Certainly we have never met with such a specimen of his wild extravagance as on the present occasion. But we have no wish to expose his absurdities any further, knowing well all the time that he is a most zealous and indefatigable physician and teacher, and firmly believing that all exclusive doctrines of medical science carry their own corrective along with them.

M. Louis, although a physician of more cautious habits and of more accurate research than his brother professor, is in our opinion not less erroneous in many of his favorite notions. He is acknowledged by all to be a most exact and admirable pathologist; but when we have said this, we have perhaps awarded him all the praise that is his due.

Judging from his writings, we have no hesitation in asserting that he is a most feeble and unscientific practitioner. In his zeal to reduce all the phenomena, operations, and effects of morbid action to certain invariable and uniform numerical laws, he loses sight of some of the most influential agents in the production and in the modification of disease. One might suppose that he had never heard of the different temperaments or diatheses of different patients; of the varying conditions of the atmosphere, as affected by climate, locality, as well as by many unknown aerial and terrestrial changes; of the influence of the mind upon the actions of the body, &c.—so complete is the omission of all such topics in most of his writings.

From the neglect of these most important enquiries in reference to the accurate examination and the judicious treatment of disease, his therapeutic directions are of little or no advantage to the practitioner, and often, as we shall presently have occasion to shew, are not only useless but are positively pernicious.

The source of all the errors of M. Louis is the vain attempt to reduce all the conclusions of medical science to the exactitude and uniformity of arithmetical calculations. He appears to consider a disease, say typhus fever for example, in nearly the same point of view, as a naturalist regards a species of animal in reference to its place in the zoological catalogue; just as if the characters or attributes of the fever were on all occasions, and under all circumstances, as uniform and steady as those of the animal. Misled by this most fallacious prejudice, it seems never to have entered into his mind that a disease, such as typhus fever, may require very different modes of treatment according to its prevailing type, and according to the constitution of the patient affected, his age, habits, and a thousand other modifying circumstances, which the experienced practitioner knows so well how to appreciate.

This is the chief excellence of the Hippocratic and Sydenham schools of medicine—the philosophic investigation that was taken by these great men and by their followers, not only of the symptoms of each case, but also of the less obvious influences and operations, which temperament, climate, age, sex, occupation, &c. exert in modifying the features and characters of different cases, as well as of different epidemics. We readily acknowledge that the physicians of these bye-gone days were greatly ignorant of correct notions on pathology; and we also most gladly admit that our improved knowledge of the morbid effects of disease has contributed not a little to introduce a more scientific and a more successful method of treatment in almost every form of those ills to which frail flesh is heir to; but, while we make these concessions in favour of our own times, we must not shut our eyes against those fallacies and errors, which have been engendered of late years by the exclusive and extravagant attention to the pathology or morbid anatomy of diseases, as if the great object of medical science was only to expound the discoveries of the dissecting-room! Hence has arisen the unnecessary, and most wearisomely tedious description of the cases narrated in most of the recent French medical works. It is not unfrequent for the details of one case to be spread over ten or a dozen pages; and often two-thirds of the whole contents of a volume are taken up with report upon report, each the exact counterpart of its predecessor.

As the leading object, which we have proposed to ourselves in this Review, is to point out wherein M. M. Louis and Bouillaud are at fault in their treatment of typhus fever, we shall not occupy much time with their descriptions of the symptoms, or of the morbid appearances of the disease. Suffice it to say that both authors seem to agree in believing that the primary and essential mischief in this disease consists in an inflammatory affection of the mucous surface of the intestines. Speaking of the anatomical lesions found in the bodies of fever patients, M. Louis uses these words:

"Of all these lesions only one was constantly found, namely, an alteration of the elliptical patches of the small intestine, to which may be added a morbid change in the mesenteric glands. I have considered it as inseparable from the disease we are now studying, and as absolutely forming its anatomical characteristic. And as it was more or less serious in some subjects who died on the eighth day of the disease; as, in the very great majority of cases, the first symptoms indicated a lesion of the alimentary canal; as the changes of the small intestine were more serious than those of the colon, which was healthy in a great number of cases, I must conclude that the lesion of the elliptical patches in the small intestine began at the commencement of the disease."

He proceeds to say:

"Although the other lesions must be considered as merely accessory or consecutive, still they commenced often quite soon after the principal disease. Especially was this the case with the different softenings, which were more serious in patients who died between the eighth and fifteenth days than in those who died afterwards." 381.

Among these accessory or consecutive lesions, by far the most frequent and important are ulcerations of the large intestine, "which," M. Louis states, "are likewise somewhat characteristic of the typhoid disease," and also enlargement and softening of the spleen. As a general summary of the pathology of typhus, we are induced to extract the following passage from M. Louis's work, more especially as it points out a most important distinction to be attended to by the morbid anatomist.

"These lesions were found more or less in all cases, were not all of the same nature, and did not all have the same cause; they were not equally frequent in patients who died at different periods of disease, and did not run their courses in the same period.

With respect to their nature, in some inflammation was more or less directly a cause; in others they appeared independent of it. Among the former were those in which there were false membranes in the pharynx; effusion of pus into the submucous cellular membrane of the pharynx, ulcerations of this organ, of the stomach and œsophagus; the mamelonated condition of the mucous membrane of the stomach; its softening in many cases; its softening with diminution of thickness in some; the more or less serious morbid changes of the elliptical patches of the ileum; that of the mesenteric glands corresponding to the patches; the softening of the lining membrane of the large intestine in many subjects; its hard patches, its ulcerations; the swelling and softening of the mesocolic glands; redness with thickening of the mucous membrane of the gall-bladder; thickening of, with redness of the pelves of the kidneys; softening of the kidneys themselves in one case; partial destructlon of the epiglottis; the first or second stages of inflammation of the lungs; false membranes of the arachnoid; finally, erysipelas and eschars upon the sacrum; ulceration with thickening of the skin in the spots where the blisters had been applied. The changes, independent of inflammation were, the pale softening of the liver and heart; the redness of the aorta, evidently at least, in the majority of the cases; the softening of the mucous membranes of the stomach, of one or both intestines in a certain number of cases; splenification of the lungs; effusion of bloody serous fluid into the pleuræ; the different conditions of the spleen; general softening of the brain, the rose or red color of the cortical substance of this organ." 379.

We are now prepared to follow M. M. Louis and Bouillaud in their description of the therapeutic means, which they have recommended in the treatment of typhus fever; and it will be interesting to the reader to notice the almost antipodal difference between the reports of the one and those of the other Parisian professor. The former, M. Louis, appears to be so sceptical of the utility of any remedial means, that we are fairly at a loss, after the perusal of his work, to state distinctly what is the treatment which he wishes us to adopt, and we are led to infer that he is quite enamoured with the advice in the Italian saying, "dolce far niente." He will, for instance, prove to you by the arithmetical comparison of a multitude of cases that nearly as many deaths occurred among those patients who were bled, as among those who were not.

Thus in one part, he says that "loss of blood had no appreciable effect upon the fatality of the disease;" in another part, that "the immediate effect of blood-letting, or that which could be observed on the day after the bleeding, is nothing, or almost nothing, or is not evident, upon the state of the symptoms of the typhoid affection; and that blood-letting, performed twice, during the first ten days of the disease, may shorten its course a little."

The admission in the last sentence of the preceding extract, viz.—"that bloodletting, performed twice, during the first ten days of the disease, *may* shorten its course a little," is the only testimony which we can draw from

M. Louis' work in favor of this remedy. He tells us that those cases, in which bleeding was performed only *once*, were quite as fatal as those in which it was not performed at all; while on the other hand he objects to a more frequent repetition of the operation than twice in any case. Indeed it is almost impossible to decide from the vague and embarrassed statements of the author what is really and truly his opinion as to the effects of this potent remedy in typhus fever, or gastro-enterite, as he calls it.

But we cannot be much surprised at this, when we call to mind that he made the important discovery (!) a few years ago, that bloodletting has very little effect on the progress and issue of certain diseases, which all physicians, Eclectics as well as the so-called Physiologists, Hippocratists, and Broussaists, must admit to be essentially inflammatory, viz. pneumonia and pleuritis !*

This is a discovery, which will indeed stagger all our preconceived notions, whether these be derived from lectures, books, or practice itself.

Were we disposed to indulge in an angry spirit of criticism, we might find abundant subject for our ire in other parts of M. Louis' Chapter on Venæsection. For example, can any British physician read without astonishment, the announcement which we have just made of our author's experience of this remedy in pneumonia or pleuritis? or of another equally important discovery (!) that "the detraction of blood, when performed for the first time and very freely between the twentieth and twenty-fifth days of a typhus fever," is injurious? But we are certain that it is quite unnecessary to express our sentiments on such practice to the British reader.

We shall therefore proceed briefly to state M. Louis' opinions on the use of tonics in fever; his remarks on this subject are very common-place and unsatisfactory. For example, all that we truly gather from several pages of perplexing description, is contained in the following few lines, which any tyro in medicine might have communicated as well as M. Louis.

"I cannot, moreover, remind the reader too often, that the conditions most favorable to the success of tonics are a moderate acceleration of the pulse, for a still stronger reason, calmness of the circulation, a natural, or *but slightly increased* heat of the skin, a respiration of little frequency, a diarrhœa which is moderate, cerebral symptoms of little intensity, or almost absent, whatever may then be the degree of debility and prostration." 447.

There is certainly no room to find fault with these directions. We wish we could say as much of the treatment of some of the reported cases, in which tonics were administered. We have marked one, the 27th, for especial reprobation, and as it is strikingly illustrative of the unscientific practice of the anatomical physicians, we shall give here a short but correct abstract of the particulars.

A youth, 17 years of age, had been indisposed for fifteen days before his admission into the hospital. Almost immediately after he was taken in, violent delirium came on, so that the use of a straight jacket was requisite. On the following day, his face was full and flushed; he was restless and agitated; there was "no stupor, but his mind was almost completely gone;"

---

* For an account of this wonderful discovery, the reader is referred to the Foreign Periscope of this Journal for July 1835.

the pupils were dilated; the abdomen was swollen and painful on pressure; there was occasional cough, and auscultation discovered a dry sonorous râle on both sides of the chest; the pulse was regular, not large, 112; and the skin was rather hot.

An infusion of cinchona, containing a large portion of extract of this drug, was prescribed; an enema of cinchona was administered, and frictions with spirits of camphor were ordered.

Next day, the patient was violently delirious. He passed urine in bed, and was frequently muttering. The same medicines were continued.

On the 3rd day, the report announces the following symptoms: drowsiness, abdomen tympanitic; pulse small, feeble and rapid; skin soft and warm.

On the 5th day, we are told that the patient was extremely tender over the whole body, especially over the abdomen, and manifested extreme impatience when touched; the meteorism and other symptoms had not altered. The same medicines were continued, and a blister was applied to the chest. The patient died next day.

The only appearances, found on dissection, to account in M. Louis' opinion for death, were a softening of the mucous coat of the stomach, and a slightly inflamed state of the elliptical glandular patches on the inner surface of the ileum.

On attentively perusing the particulars of this case, we do not hesitate to assert that the practice, which was pursued, was most unscientific and improper. The existence of the violent delirium and of other symptoms indicative of constitutional excitement, which were present on the patient's admission into the hospital, would have induced a British physician to have abstained from giving large doses of cinchona. Instead of such treatment, he would have ordered the patient's head to be shaved, and kept cold with a spirituous lotion, and, if the vital powers did not authorize cautious depletion by means of leeches or the cupping glasses, he would most certainly have prescribed such remedies as effervescing draughts,* or other saline refrigerants.

When the proper period for administering tonics had arrived, he would prefer many others to large doses of extract of cinchona. The carbonate of

---

* The French physicians seem to be quite ignorant of this most common and useful form of medicine. We never meet with such a prescription as the haust. salin. effervescens in any of their writings; and we were not a little amused by reading the following remarks from such a well-informed author as M. Louis: "The English have, of late, revived the use of the carbonic acid, which was formerly of repute in the treatment of the typhoid affection. Nine patients, affected with this disease, have been very recently treated by M. Chomel in the wards of La Charité with gaseous water in enemata and in drinks. All of them, two of whom experienced severe symptoms (prolonged somnolency, delirium and meteorism) recovered. Another patient, treated in the same manner, in the city, by the same physician, died. If this first experiment cannot decide the question of the utility or inutility of the carbonic acid, it may induce us to administer it whenever the occasion presents itself." Such is a specimen of the utter ignorance of our neighbour as to the principles and practice of British medicine!

ammonia in an aromatic infusion, the mineral acids, with or without the use of wine or brandy, are among the safest and best.

The only other remedies which M. Louis treats of, are blisters to the neck or lower extremities, and the use of ice upon the head. With reference to the former, our author states:

"Blisters ought to be banished from the treatment of the typhoid affection, and with the more reason, as every one knows their bad effects, the loss of substance which they occasion, and the slowness with which their wounds heal in many cases. While they are of no utility towards the re-establishment of the cerebral functions, they concur, however, in maintaining or augmenting the febrile action and its troublesome consequences; their effect, as a derivative means for many of the inflammations which manifest themselves in the course of the typhoid affection, is more than doubtful, after what we have just seen above, an inflammation of a small extent in an organ almost inevitably bringing one or many others in its train. So that whatever view we take of blisters, we find only inconveniences, without any advantages to counterbalance them." 441.

And as to the application of ice to the head, it is condemned as equally inefficacious and improper:

"Moreover, if blisters had no appreciable influence upon the delirium and the somnolency, the same was the case with the application of ice upon the head and leeches to the neck, alone or in conjunction; so that we do not see why we should recommend their employment against the delirium, whatever may be its degree." 449.

Such is the sum and substance of M. Louis' therapeutic instructions in reference to typhus fever—a disease, than which none in the nosological catalogue requires, for its scientific and successful treatment, greater tact, discrimination, and delicacy. It is not by pursuing in all epidemics, or even in all cases of the same epidemic, a uniform, exclusive, and undeviating mode of practice—as, we fear, is far too much the case with the French physicians—that we can ever hope to be extensively fortunate. Bleeding and blistering may be required in some cases, and the use of tonics and of ice to the head may be demanded in others; but at the same time we can assure M. Louis that neither the one nor the other line of treatment is well suited to a very large proportion.

It is of especial importance in the management of certain forms of typhus fever that the practitioner be not over-officious, either with the use of his lancet and leeches, or with the exhibition of his Peruvian bark and his wine.

The cautious use of aperients—and the only safe guide in the use of these is a daily inspection of the alvine evacuations, as recommended by Dr. James Hamilton in his admirable work on purgatives—the administration of the milder mercurials, either alone or in combination with James' powder, or with mild refrigerants, such as the nitrate of potass, and the use of saline diaphoretics in the state of effervescence, constitute by far the most successful treatment in a multitude of cases of typhoid fever. The judicious practitioner will never omit to pay a due attention to the cutaneous surface, as well as to the state of all the mucous linings. For this purpose sponging the skin of the body and limbs with tepid water and vinegar twice a day will be found most beneficial, and certainly most soothing and refreshing to the patient's feelings. The French physicians seem to be little acquainted with the good effects of this simple remedy.

But we must hasten to say a few words on the practice of M. Bouillaud, before closing. As a matter of course, bleeding is with him the grand remedy. The quantity of the blood drawn, and the frequency of the operation are, he admits, to vary with the gravity of the existing symptoms; but in no case, or at least in exceedingly few, is venesection to be omitted.

The average quantity drawn in the cases treated by M. Bouillaud was two pounds, or thereabouts. The mortality did not exceed one in eight; whereas, according to our author, in the practice of M. M. Chomel, Louis and others, who adopt a less vigorous practice, it amounts to one in three or four. Certainly this would be decisive, if the data were satisfactory; but, as we have remarked above, we have strong reason to suspect that M. Bouillaud classes numerous cases in his catalogue of typhoid fever, which the British physician, and perhaps also many of his countrymen, might regard as of a much more simple character.

The other remedies mentioned by M. Bouillaud, as having been used by him, are a low diet and refrigerant drinks, such as lemonade, iced water, or a solution of any pleasant vegetable lymph; enemata, baths, or affusions, ice to the head and to the abdomen, blisters and sinapisms to the limbs or to the abdomen; and lastly musk, charcoal and the chlorurets.

He tells us that aperients are rarely required, and that the very mildest only should be used. He has devoted a chapter to the consideration of the treatment of typhoid fever "par la methode purgative nouvelle."* The *novelty* of this method consists apparently in purging the patient to death's door, without any regard to the state of the alvine evacuations, or to the existing symptoms of the disease. M. Bouillaud has related three cases, in which the patients very speedily died, under the use of purgatives administered "*coup sur coup.*" Mark the phrase "*coup sur coup.*" It at once reveals the absurd and unscientific principles on which these schoolmen proceed, in the treatment of so serious a disease as typhus fever. Throughout the chapter there is not even one passing allusion made to the work of Dr. Hamilton, or of any other British physician. So much for the author of a work on the philosophy of medical science in the nineteenth century.

---

* The reader will find, in the Foreign Periscope of the present number, the report of an interesting discussion on the treatment of typhoid fever by the use of purgatives, which recently took place at the Royal Academy of Medicine in Paris.

M. Andral introduced the subject, and many of the most eminent metropolitan physicians took part in the debate. We strongly recommend our readers to peruse this article.—*Rev.*

